# Sex-specific microRNA expression networks in an acute mouse model of ozone-induced lung inflammation

**DOI:** 10.1186/s13293-018-0177-7

**Published:** 2018-05-08

**Authors:** Nathalie Fuentes, Arpan Roy, Vikas Mishra, Noe Cabello, Patricia Silveyra

**Affiliations:** 1 Pulmonary, Immunology and Physiology Laboratory, Department of Pediatrics, The Pennsylvania State University College of Medicine, 500 University Drive, H085, Hershey, PA 17033 USA; 2 Department of Life Sciences, Presidency University, Kolkata, India; 3 Department of Biochemistry and Molecular Biology, The Pennsylvania State University College of Medicine, Hershey, PA USA

**Keywords:** Lung miRNome, Estrous cycle, Air pollution, miR-712-5p, miR-106a-5p

## Abstract

**Background:**

Sex differences in the incidence and prognosis of respiratory diseases have been reported. Studies have shown that women are at increased risk of adverse health outcomes from air pollution than men, but sex-specific immune gene expression patterns and regulatory networks have not been well studied in the lung. MicroRNAs (miRNAs) are environmentally sensitive posttranscriptional regulators of gene expression that may mediate the damaging effects of inhaled pollutants in the lung, by altering the expression of innate immunity molecules.

**Methods:**

Male and female mice of the C57BL/6 background were exposed to 2 ppm of ozone or filtered air (control) for 3 h. Female mice were also exposed at different stages of the estrous cycle. Following exposure, lungs were harvested and total RNA was extracted. We used PCR arrays to study sex differences in the expression of 84 miRNAs predicted to target inflammatory and immune genes.

**Results:**

We identified differentially expressed miRNA signatures in the lungs of male vs. female exposed to ozone. In silico pathway analyses identified sex-specific biological networks affected by exposure to ozone that ranged from direct predicted gene targeting to complex interactions with multiple intermediates. We also identified differences in miRNA expression and predicted regulatory networks in females exposed to ozone at different estrous cycle stages.

**Conclusion:**

Our results indicate that both sex and hormonal status can influence lung miRNA expression in response to ozone exposure, indicating that sex-specific miRNA regulation of inflammatory gene expression could mediate differential pollution-induced health outcomes in men and women.

**Electronic supplementary material:**

The online version of this article (10.1186/s13293-018-0177-7) contains supplementary material, which is available to authorized users.

## Background

Ground-level ozone (O_3_) is a reactive oxidant gas that is a major constituent of air pollution [1]. Ozone is formed by the photochemical reactions of carbon monoxide, nitrogen oxides, and chemically active hydrocarbons also known as volatile organic compounds and mostly occurs downwind of major cities. The association of short-term ambient exposure of O_3_ with the incidence of respiratory afflictions such as asthma, idiopathic pulmonary fibrosis, and chronic obstructive pulmonary disease (COPD), as well as cardiovascular mortality indicates that O_3_ is a powerful toxicant for the cardiorespiratory system [2–6]. In addition, epidemiological studies have reported sex differences in the incidence and prognosis of pollution-induced respiratory diseases and have shown that women are at increased risk of adverse health outcomes from O_3_, particulate matter, and cigarette smoke exposure than men [7–10].

Being a gaseous pollutant, the primary effect of O_3_ occurs in the lung causing a range of respiratory ailments [11–13]. The mechanisms by which O_3_ mediates these effects involve generation of reactive oxygen species (ROS) triggering oxidative stress [14]. In addition, pro-inflammatory cytokines have been implicated as potential mediators of lung oxidative injury in response to air pollution exposure [15]. Among these cytokines, interleukin-6 (IL-6) contributes to the initiation and extent of the inflammatory process [16]. In a previous study, we have demonstrated that expression of IL-6 in the lung is significantly induced by O_3_ inhalation in both males and females, with significantly higher levels in females vs. males [17]. However, to this day, the molecular mechanisms involved in the observed sex differences remain unknown.

In the last couple of decades, a novel post-transcriptional gene regulation machinery has been identified with the discovery of short (19–25 nucleotides), naturally occurring, non-coding RNA molecules, known as microRNAs (miRNAs). This class of small RNA molecules is evolutionarily conserved and functions in the fine-tuning of gene expression by direct translational inhibition and/or induction of target mRNA degradation [18]. It has also been reported that miRNAs can be oxidized in response to oxidative stress, via guanine hydroxylation, altering their ability to bind to target mRNA sequences [19]. In addition, miRNAs are involved in various important biological processes such as the immune response, cell differentiation, developmental processes, and apoptosis [20, 21]. In the lung, miRNAs play important roles in developmental processes and in homeostasis maintenance, and their abnormal expression has been associated with the development and progression of various pulmonary diseases [22–25].

The role of miRNAs in lung development was first elucidated in mice, where conditional deletion of Dicer (an important enzyme of the miRNA synthesis pathway) in lung epithelial cells resulted in impaired epithelial branching and developmental abnormalities and also led to dysregulated cell death [26]. In addition, abnormal expression of miRNAs has been correlated with the occurrence of pulmonary disorders such as asthma, COPD, and lung cancer in both children and adults [27–31]. Despite the known sex disparities in the incidence and severity of these diseases [32, 33], there are currently very few studies exploring the role of miRNAs in mediating these sex-biased disease outcomes [34].

We have previously reported sex differences in the expression of lung inflammatory markers in response to O_3_, and we have shown that pre-exposure to this air pollutant affected lung immunity in a sex-specific manner [35–37]. Additional studies revealed a potential role of gonadal hormones in this regulation [38]. However, the molecular mechanisms by which the male and female lungs respond to ambient O_3_, and the specific role of miRNAs in this regulation, have not yet been explored. Based on these preliminary data, we hypothesized that sex-specific miRNA expression can mediate gender-specific immune responses to O_3_ via modulation of pulmonary inflammatory gene expression. Thus, the goal of this study was to determine whether sex and hormonal status could modulate lung miRNA expression networks during O_3_-induced acute inflammation. For this, we compared the expression of specific miRNAs in the lungs of male and female mice exposed to O_3_ or filtered air (FA, control), and we used bioinformatics approaches to compare predicted regulatory networks and target genes associated with innate immunity and inflammation. With the goal of evaluating potential contributions of female sex hormones to these networks, we also evaluated differences in the lung miRNA expression of female mice exposed to O_3_ or FA at different stages of the estrous cycle. Our results indicate that O_3_ exposure differentially affects lung miRNA expression in male and female mice and that the stage of the estrous cycle does affect the miRNA expression signature. We also identified miRNAs that have been previously associated with IL-6 regulation and that were differentially expressed in females and males in response to O_3_ challenge [39, 40]. To our knowledge, this is the first study investigating both inflammatory miRNA networks and hormonal influences in response to O_3_ exposure. This information can have significant implications for environmental and women’s health and the development of novel therapeutics to treat and prevent lung disease in women.

## Methods

### Animals

Adult male and female mice (8 weeks of age) from the C57BL/6 background were purchased from JAX laboratories (Bar Harbor, ME) and housed and maintained in a 12/12-h light/dark cycle with food and water available ad libitum. The Pennsylvania State University College of Medicine Institutional Animal Care and Use Committee (IACUC) approved all procedures (protocol #42135). The institution is accredited by the Association for Assessment and Accreditation of Laboratory Animal Care (AAALAC).

### Assessment of estrous cycle stage

We determined estrous cycle stage in female mice by analysis of daily vaginal secretions for at least three consecutive cycles, as described previously [41]. For this, a smear of vaginal flush was prepared and observed under light microscope. Based on the smear appearance, the estrous cycle stage was determined by the proportion of nucleated epithelial cells, leukocytes, and cornified cells, as follows: proestrus (predominantly nucleated epithelial cells), estrus (predominantly anucleated cornified cells), diestrus 1/metestrus (all three types of cells), and diestrus 2 (majority of leukocytes). Animals that did not show regular cycles due to pseudopregnancy or other causes were excluded from the experiment.

### Exposure to O_3_

Male mice and female mice at different stages of the estrous cycle (*n* = 3–9 animals per group) were placed in glass containers with wire mesh lids containing bedding, food, and water ad libitum. Nest packs were also provided to the experimental animals to provide enrichment material to promote normal behavior and limit the extent of stress and fighting. On the day of the experiment, mice were exposed to 2 ppm of O_3_ for 3 h, using an exposure chamber (2.089 ± 0.021 ppm). [42]. Control animals were exposed to filtered air (FA) for 3 h in an adjacent chamber. The apparatus delivers a regulated air flow (> 30 air changes/hour) with controlled temperature (25 °C) and relative humidity (50%). Following exposure, animals were removed from the apparatus, and samples were collected as described below. At 4 h after exposure, animals were anesthetized with an intraperitoneal injection of a ketamine/xylazine cocktail (90 mg/kg ketamine, 10 mg/kg xylazine). A midline incision was made, and blood was collected by aspiration from the inferior vena cava. Mice were then euthanized by transection of the vena cava and aorta. Total lung tissue was collected and snap frozen in liquid nitrogen for miRNA expression experiments. To control for any circadian variations and to be able to monitor evening hormone peaks associated with estrous cycle stages, we exposed all animals at the same time of the day (11:00 am–2:00 pm) regardless of cycle day. The lungs and blood were harvested at 6:00 pm. The concentration of O_3_ used in this study is higher than that normally found in the atmosphere. The rationale for using this concentration is that higher doses are required for rodents vs. humans to reach comparable O_3_ concentrations in the distal lung [43] and that rodents acutely exposed to 2 ppm of O_3_ show comparable or lower levels of inflammatory markers than exercising humans exposed to much lower concentrations (0.4 ppm) [44].

### Serum hormone determinations

To verify the estrous cycle stage in females, serum levels of estradiol and luteinizing hormone were determined by ELISA (cats. #MBS9424676 and #MBS041300, MyBioSource, San Diego, CA).

### RNA preparation

Total RNA was extracted from pulverized tissue using Trizol and the Direct-Zol RNA extraction kit (Zymo Research), following the manufacturer’s instructions. Total RNA concentration was measured by Nanodrop, and RNA quality was confirmed by Bioanalyzer as indicated by RIN > 7 at the Pennsylvania State University College of Medicine Genome Sciences Core Facility.

### miRNA profiling

Small RNAs were retro-transcribed from 200 ng of total RNA using the miScript II RT kit (Qiagen). The expression of 84 mouse miRNAs predicted to regulate inflammatory genes was assayed with the Mouse Inflammatory Response and Autoimmunity miRNA PCR Array (MIMM-105Z, Qiagen). A list of miRNAs and predicted targets can be found at https://www.qiagen.com/us/shop/pcr/primer-sets/miscript-mirna-pcr-arrays/?catno=MIMM-105Z#geneglobe.

### Data analysis

Results were analyzed using the QuantStudio 12K Flex Software, and Ct values were exported to MS excel. Data were processed following recommendations described in studies using similar samples. Briefly, data were analyzed in excel using Ct values for each sample, normalized to the average Ct of five miRNA housekeeping miRNAs controls (SNORD61, SNORD68, SNORD72, SNORD95, SNORD96A, RNU6-2) as ΔCt = (Ct_Target − Ct_housekeeping). For fold change calculations, ΔΔCt-based fold-change values were obtained using sample 27 as control, using the Livak method (2^− ΔΔCt^, where − ΔΔCt = − [ΔCttest − ΔCtcontrol]) [45]. Arrays shown in figures are representative of fold changes calculated with this method. Statistical analyses were performed with the R software using the Bioconductor limma package to detect differences among treatments and correcting for multiple comparisons using the Benjamini-Hochberg method [46]. Differential expression was defined as a Benjamini-Hochberg false discovery rate (FDR) of less than 0.05.

### Ingenuity pathway analysis

Significantly altered transcripts from analysis with PCR arrays were used as input for the miRNA Target Filter function in Ingenuity Pathway Analysis (IPA, Qiagen Redwood City, https://www.qiagenbioinformatics.com/products/ingenuity-pathway-analysis/) to find predicted miRNA-regulated target genes differentially expressed in the lungs of males and females exposed to O_3_. Using the premise that reciprocal expression patterns exist between miRNA and their predicted gene targets within the defined list of differentially expressed genes, networks of predicted miRNA-regulated genes were constructed to visualize the potential effects of individual miRNAs on networks. We used the IPA miRNA Target Filter function, which incorporates experimentally demonstrated and in silico predicted miRNA-mRNA interactions from the databases TargetScan, TarBase, and miRecords. IPA was used to perform functional gene enrichment analysis using predicted target genes from miRNA-centered networks. Correlation of expression patterns of miRNAs and differentially expressed transcripts were performed with logarithmic fold changes and *P* values.

## Results

### Sex differences in basal miRNA expression

Previous investigations have documented differences in pulmonary function parameters, innate immune responses, and lung disease pathogenesis in female and male mice breathing clean air. With a few exceptions, male mice are usually characterized by weaker immune responses than female mice [47, 48]. In our model, the miRNA expression array data in lung tissue acquired from mice exposed to filtered air showed differences in miRNA expression between males and females (Additional file 1: Figure S1A). Two miRNAs, miR-222-3p and miR-466 k, were differentially expressed. MicroRNA-222-3p and miR-466k were upregulated (log fold change = 0.459) and downregulated (log fold change = − 0.614), respectively, in males vs. females (Additional file 1: Figure S1B). The in silico analysis showed a relationship between these miRNAs and major gene families such as transcription factors and proto-oncogenes (FOS, JUN, FOXO3, FOXP3, E2F1, CDKN2B, CCND1, ARID3B, TP53, KIT), translation regulators (AGO2), transporters (vesicle-mediated transporter CLVS2, channel/pore class transporter BCL2), nuclear receptors (ESR1, RORB), kinases (BRAF, SBK1), growth factors (BDNF), phosphatases (PTEN), and proteins in the extracellular matrix (TIMP3) (Additional file 2: Table S1). IPA analysis revealed that these molecules are associated with top molecular functions such as cell-to-cell signaling and interaction, cellular growth, proliferation, and gene expression (Additional file 3: Figure S2A).

### Sex differences in O_3_-induced lung miRNA expression

**Fig. 1:**
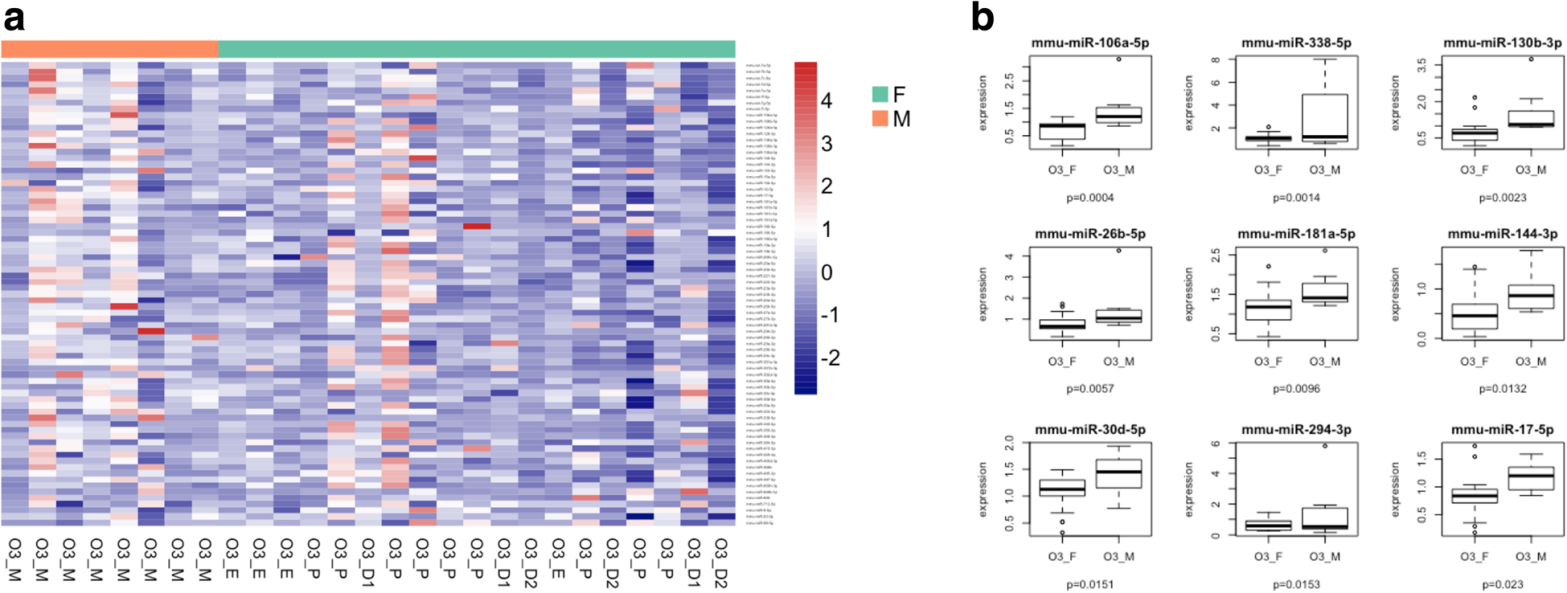
Sex differences in O_3_-induced lung miRNA expression. **a** Cluster analysis of 84 miRNAs expressed in lung extracts obtained from male and female mice following exposure to 2 ppm of O_3_ for 3 h. **b** Differentially expressed miRNAs in male and female lung extracts. Results are expressed as female miRNA expression levels relative to male miRNA expression levels. M males (*n* = 8), F females (*n* = 19), O3_M males exposed to ozone, O3_D1 females exposed to ozone in diestrus 1, O3_D2 females exposed to ozone in diestrus 2, O3_P females exposed to ozone in proestrus, O3_E females exposed to ozone in estrus

The screening of miRNA expression in the lungs of male and female mice exposed to O_3_ allowed the detection of miRNAs differentially expressed between these two groups. Further analysis performed with IPA revealed that the top molecular functions associated with differentially expressed genes in males vs. females exposed to O_3_ were linked to cell cycle, cellular development, and cellular growth and proliferation, which are important pathways in the lung inflammatory response. Moreover, the top associated network functions included organismal and tissue development, humoral immune response, nervous system development, and reproductive system development and function. Several of these were also involved in inflammation (miR-130b-3p, miR-17-5p, miR-294a-3p, and miR-338-5p) and targeted key regulators of the immune response including IL-6, SMAD2/3, and TMEM9 (Table 1, Additional file 3: Figure S2B). In total, there were nine miRNAs whose expression was significantly lower in females vs. males exposed to O_3_ (Fig. 1).

**Table 1:** Target genes and associated regulatory networks for differentially expressed miRNAs in lung tissue of male and female mice exposed to ozone

A. Genes targeted by differentially expressed miRNAs					
BCL2 CDKN1A COX8C DDHD1	ENPP5 FBXO48 FGD4 FICD	GPR158 GPR137C IL6 MARCH4	MBNL2 MYT1L PCNX1 PTHLH	RDH14 RP11_65D242 SLITRK3 Smad2/3	SMAD6/7 TMEM9B ZNF800
B. Differences in top diseases and biofunctions					
Diseases and disorders				*P* value	
Cancer				4.80E−02 to 4.62E−11	
Organismal injury and abnormalities				4.80E−02 to 4.62E−11	
Reproductive system disease				4.01E−02 to 4.62E−11	
Endocrine system disease				2.28E−02 to 3.11E−07	
C. Top molecular and cellular functions					
Molecular and cellular functions				*P* value	
Cell cycle				1.23E−02 to 1.87E−05	
Cellular development				4.05E−02 to 5.81E−05	
Cellular growth and proliferation				3.76E−02 to 5.81E−05	
D. Top physiological system development and function					
Development and function				*P* value	
Organismal development				3.76E−02 to 1.11E−05	
Tissue development				3.76E−02 to 7.49E−04	
Reproductive system development and function				1.04E−02 to 2.99E−03	
E. Top associated network functions					
Associated network functions				Score	
Cancer, organismal injury, and abnormalities				27

### Differential regulatory pathways are activated in males vs. females in response to O_3_

**Fig. 2:**
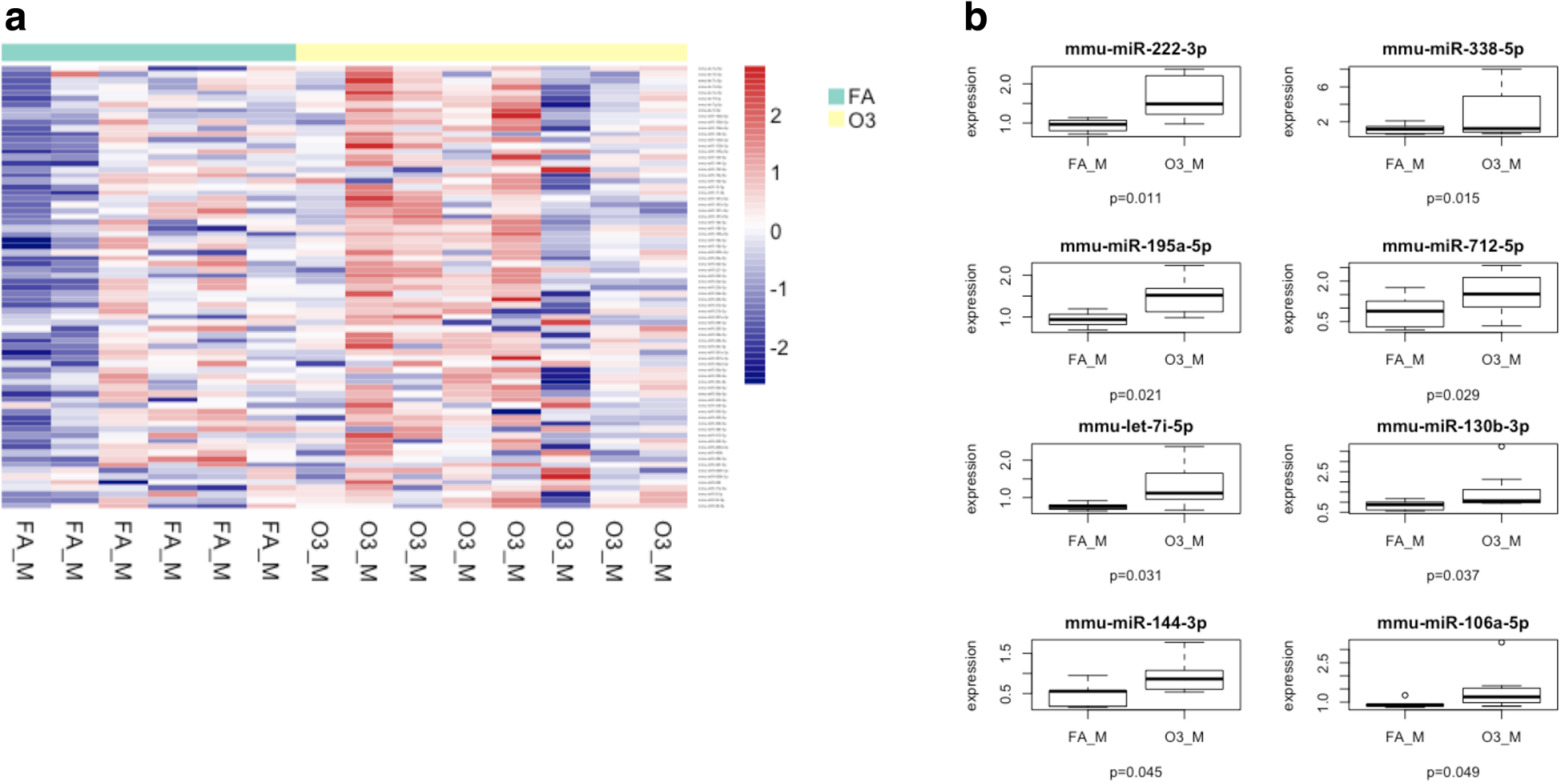
Expression of lung miRNAs in males exposed to O_3_ vs. FA. **a** Cluster analysis of 84 miRNAs in lung extracts from male mice exposed to 2 ppm of O_3_ or FA for 3 h. **b** Differentially expressed miRNAs in males exposed to O_3_ vs FA. FA filtered air, O3 ozone, FA_M males exposed to filtered air (*n* = 6), O3_M males exposed to ozone (*n* = 8)

**Fig. 3:**
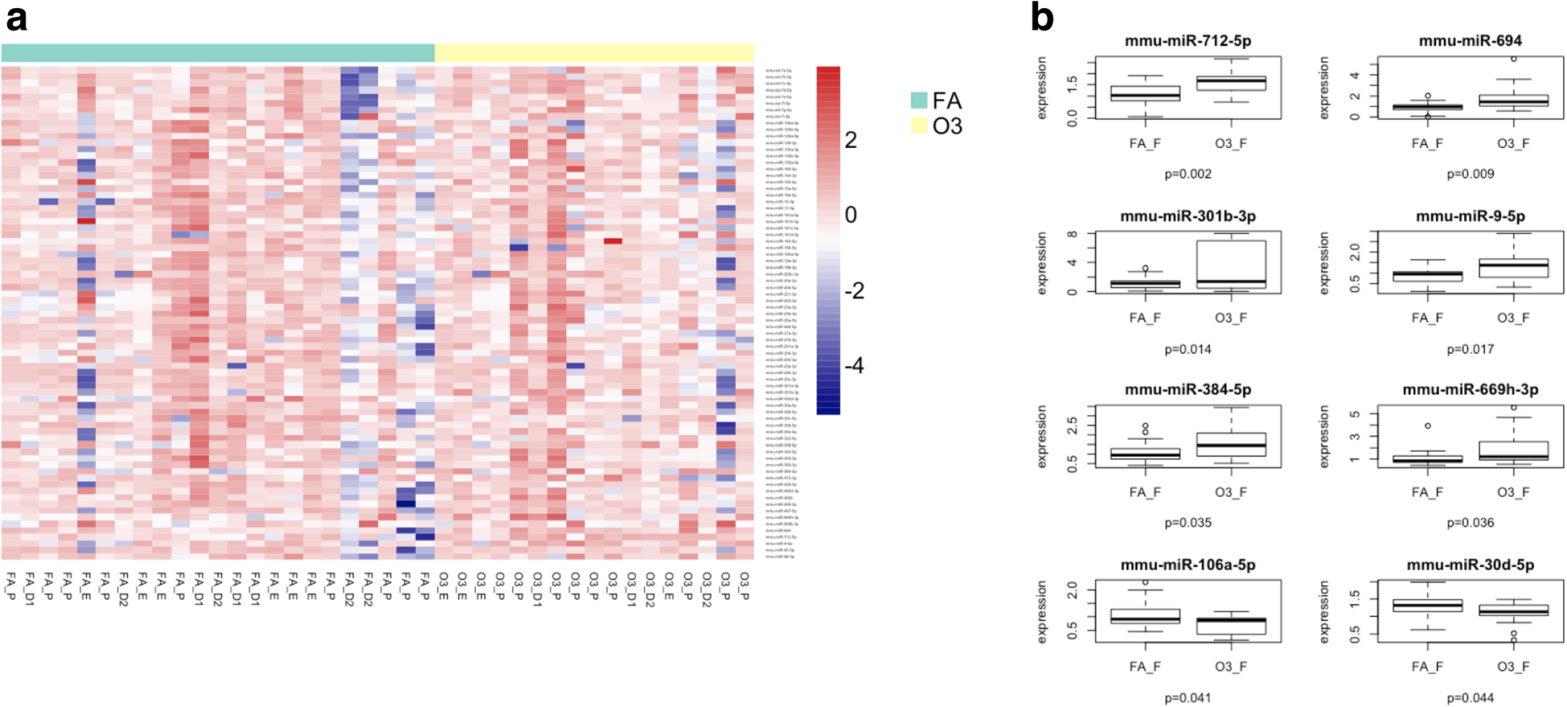
Expression of lung miRNAs in females exposed to O_3_ vs. FA. **a** Cluster analysis of 84 inflammatory miRNAs in lung extracts from female mice exposed to 2 ppm of O_3_ or FA for 3 h. **b** Differentially expressed miRNAs in females exposed to O_3_ vs FA. FA filtered air (*n* = 23), O3 ozone (*n* = 19), FA_D1 females exposed to filtered air in diestrus 1, FA_D2 females exposed to filtered air in diestrus 2, FA_P females exposed to filtered air in proestrus, FA_E females exposed to filtered air in estrus, O3_D1 females exposed to ozone in diestrus 1, O3_D2 females exposed to ozone in diestrus 2, O3_P females exposed to ozone in proestrus, O3_E females exposed to ozone in estrus

Next, we analyzed sex differences in the miRNA response to O_3_ vs. FA exposure. Of the eight differentially expressed miRNAs found in both males and females exposed to O_3_, a total of six miRNAs were upregulated exclusively in males: miR-338-5p (log fold change = 1.636), miR-222-3p (log fold change = 0.699), miR-130b-3p (log fold change = 0.646), let-7i-5p (log fold change = 0.552), miR-195a-5p (log fold change = 0.543), and miR-144-3p (log fold change = 0.427) (Fig. 2). IPA analysis revealed that the top cellular functions associated with these miRNAs and their targets were cell cycle, cell death, cell survival, and cellular movement. The top interaction networks for these miRNAs were related to digestive system development and function, gastrointestinal disease, hepatic system development and function, and inflammatory disorders and response (Table 2). In females, O_3_ exposure induced the expression of miR-301b-3p (log fold change = 1.652), miR-694 (log fold change = 0.727), miR-669 h-3p (log fold change = 0.679), miR-384-5p (log fold change = 0.455), and miR-9-5p (log fold change = 0.378) and downregulated the expression of miR-30d-5p (log fold change = − 0.204) (Fig. 3). Some of these miRNAs target important regulators of the immune system such as SOCS5 and IL-10RB, which may be altering the lung host defense (Table 2). The top interaction networks in females exposed to O_3_ were associated with cancer, organismal injury and abnormalities, and reproductive system disease. The top molecular functions affected were cellular development, cellular growth, proliferation, and cell cycle.

**Table 2:** Summary obtained from IPA analysis of FA and O_3_ exposed male and female mice

Males			Females		
A. Genes targeted by differentially expressed miRNAs					
ARHGEF38 Cg CPEB2 CPEB3 ENTPD7 EPHB6 FAM35A GAREM1 GPR63 Gulo	IL6 KIAA1328 MIGA1 NT5DC1 PLAT RFX7 SECISBP2L SKIDA1	SLC38A1 TMCC1 TMEM110 MEM55A TSPAN13 UTS2B XKR8 ZCCHC11	APCDD1 ARHGAP12 BTG1 C2orf15 CMTR2 COX8C DYNC1LI2 ENPP5 FBXO48	FICD Gulo IL10RB INSR MBNL1 PAPD4 PLSCR4 PXK RP11_65D242	RRAGD SLC10A3 SMOC1 SNX2 SOCS5 STIM2 STX6 TMEM170B ULK2
B. Differences in top diseases and biofunctions					
Diseases and disorders		*P* value	Diseases and disorders		*P* value
Gastrointestinal disease		4.98E−02 to 3.39E−07	Cancer, organismal injury and abnormalities		4.85E−02 to 2.22E−10
Inflammatory disease		4.06E−02 to 3.39E−07	Reproductive system disease		3.24E−02 to 2.22E−10
Inflammatory response		4.06E−02 to 3.39E−07	Endocrine system disease		3.84E−02 to 2.22E−06
C. Top molecular and cellular functions					
Molecular and cellular functions		*P* value	Molecular and cellular functions		*P* value
Cell cycle		1.26E−02 to 3.46E−05	Cellular development		4.06E−02 to 2.54E−04
Cell death and survival		4.95E−02 to 8.00E−05	Cellular growth and proliferation		4.06E−02 to 2.54E−04
Cellular movement		4.11E−02 to 9.68E−05	Cell morphology		1.10E−02 to 5.62E−04
D. Top physiological system development and function					
Development and function		*P* value	Development and function		*P* value
Digestive system development and function		1.51E−02 to 3.39E−07	Organismal development		4.85E−02 to 2.22E−10
Hepatic system development and function		1.51E−02 to 3.39E−07	Endocrine system disorders		3.84E−02 to 2.22E−06
Organ development		4.98E−02 to 3.39E−07	Reproductive system development and function		3.24E−02 to 2.22E−10
E. Top associated network functions					
Associated network functions		Score	Associated network functions		Score
Digestive system development and function, gastrointestinal disease, hepatic system development and function		17	Cancer, organismal injury and abnormalities, reproductive system disease		14

**Fig. 4:**
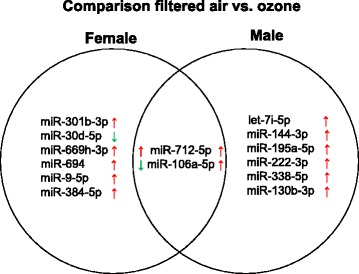
Venn diagram of differentially expressed lung miRNAs in male and female mice exposed to O_3_ vs. FA. Arrows indicate upregulation (red) or downregulation (green) of miRNA expression in lungs tissue of O_3_-exposed vs. FA-exposed mice

**Fig. 5:**
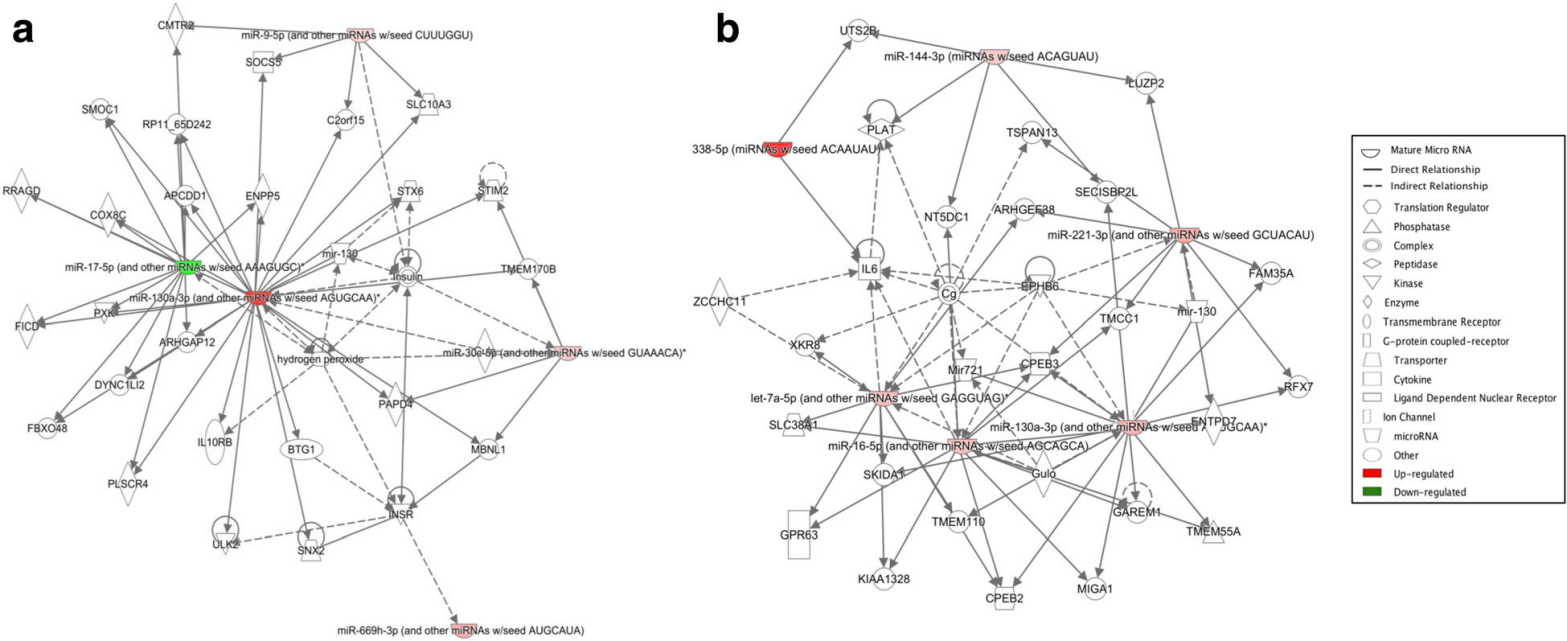
Differential regulatory networks activated in female and male mice exposed to O_3_ vs. FA. Diagram of networks of miRNAs whose expression was affected by O_3_ exposure in female (**a**) or male (**b**) mice, generated by IPA. Both diagrams show reported direct (solid lines) and indirect (dashed lines) interactions. Molecules that are downregulated or upregulated are represented as a node in green or red, respectively

Interestingly, two miRNAs were affected by O_3_ exposure in both males and females (Fig. 4). Of these, miR-712-5p was the only miRNA found upregulated in both males (log fold change = 0.658) and females (log fold change = 0.543). Interestingly, miR-106a-5p was upregulated in males (log fold change = 0.502) but downregulated in females (log fold change = − 0.302) following O_3_ exposure. Several genes essential for the lung inflammatory response were predicted to be targeted by these miRNAs (Tables 3 and 4). Comparison of the main pathways affected by O_3_ also confirmed differentially affected functions in males vs. females. Some miRNAs such as miR-338-5p, miR-106a-5p, and let-7a-5p (affected exclusively in males) were predicted to target the IL-6 family both directly and indirectly (Fig. 5).

**Table 3:** Serum levels of luteinizing hormone (LH), estradiol (E2), and progesterone (P4) at the time of sample collection (6:00 pm) in female mice at different stages of the estrous cycle

	LH (mIU/mL)		E2 (pg/mL)		P4 (ng/mL)	
	Mean	SEM	Mean	SEM	Mean	SEM
Metestrus	5.66	0.19	3.77	0.35	1.99	0.20
Diestrus	4.96	0.33	2.59	0.38	5.89	1.84
Proestrus	9.78*	0.43	7.75*	0.55	4.08	0.67
Estrus	5.79	0.44	3.74	0.39	2.49	0.37

*Significant difference vs. all other stages, *P* < 0.0001

**Table 4:** IPA summary of females exposed to ozone in the non-proestrus and proestrus stages

Non-proestrus			Proestrus		
A. Genes targeted by differentially expressed miRNAs					
CAMK2N1 CARS CYP24A1 DBF4 HMGN2 KMT5A LRRC17 MDH2 MIS18A	PAFAH1B2 PDE4B PDE7A PGM3 PNP REV1 RPS19BP1 RRAD SEC62	SEC23A SNX5 SYT4 THAP12 TMED7 TNFAIP2 TNFRSF10C TP53 UBE2V2 ZNF420	ABCB9 APOO ARHGEF38 AVEN CASP3 CCNJ CMTR2 CNMD DSCR8	FGD4 FRMD4B GPR137B HMBS METAP1 MITF MYC RGMB SLC14A1	SLC25A27 SLC38A1 TMCC1 TRIM71 XKR8 ZCCHC11 ZIM3 ZNF181
B. Differences in top diseases and biofunctions					
Diseases and disorders		*P* value	Diseases and disorders		*P* value
Inflammatory disease		3.84E−02 to 3.84E−05	Organismal injury and abnormalities		4.96E**−**02 to 2.77E**−**14
Inflammatory response		3.84E**−**02 to 3.84E**−**05	Reproductive system disease		2.15E**−**02 to 2.77E**−**14
Organismal injury and abnormalities		4.17E**−**02 to 4.17E**−**05	Cancer		4.96E**−**02 to 1.27E**−**10
C. Top molecular and cellular functions					
Molecular and cellular Functions		*P* value	Molecular and cellular functions		*P* value
Cellular development		2.05E**−**02 to 5.26E**−**07	Cellular movement		3.77E**−**02 to 4.47E**−**07
Cellular compromise		3.75E**−**04 to 3.75E**−**04	Cellular death and survival		4.91E**−**02 to 5.61E**−**06
Cell cycle		2.62E**−**03 to 2.62E**−**03	Cellular development		4.97E**−**02 to 1.38E**−**06
D. Top physiological system development and function					
Development and function		*P* value	Development and function		*P* value
Organismal development		4.17E**−**02 to 1.31E**−**03	Embryonic development		3.30E**−**02 to 2.12E**−**05
Embryonic development		1.29E**−**02 to 1.29E**−**02	Connective tissue development and function		1.79E**−**02 to 6.10E**−**05
Connective tissue development and function		1.93E**−**02 to 1.93E**−**02	Tissue morphology		7.88E**−**05 to 7.88E**−**05
E Top associated network functions					
Associated network functions		Score	Associated network functions		Score
Cellular development, inflammatory disease, inflammatory response		6	Organismal injury and abnormalities, reproductive system disease, cancer		19

### Effect of the estrous cycle stage in the miRNA response to O_3_ exposure

**Fig. 6:**
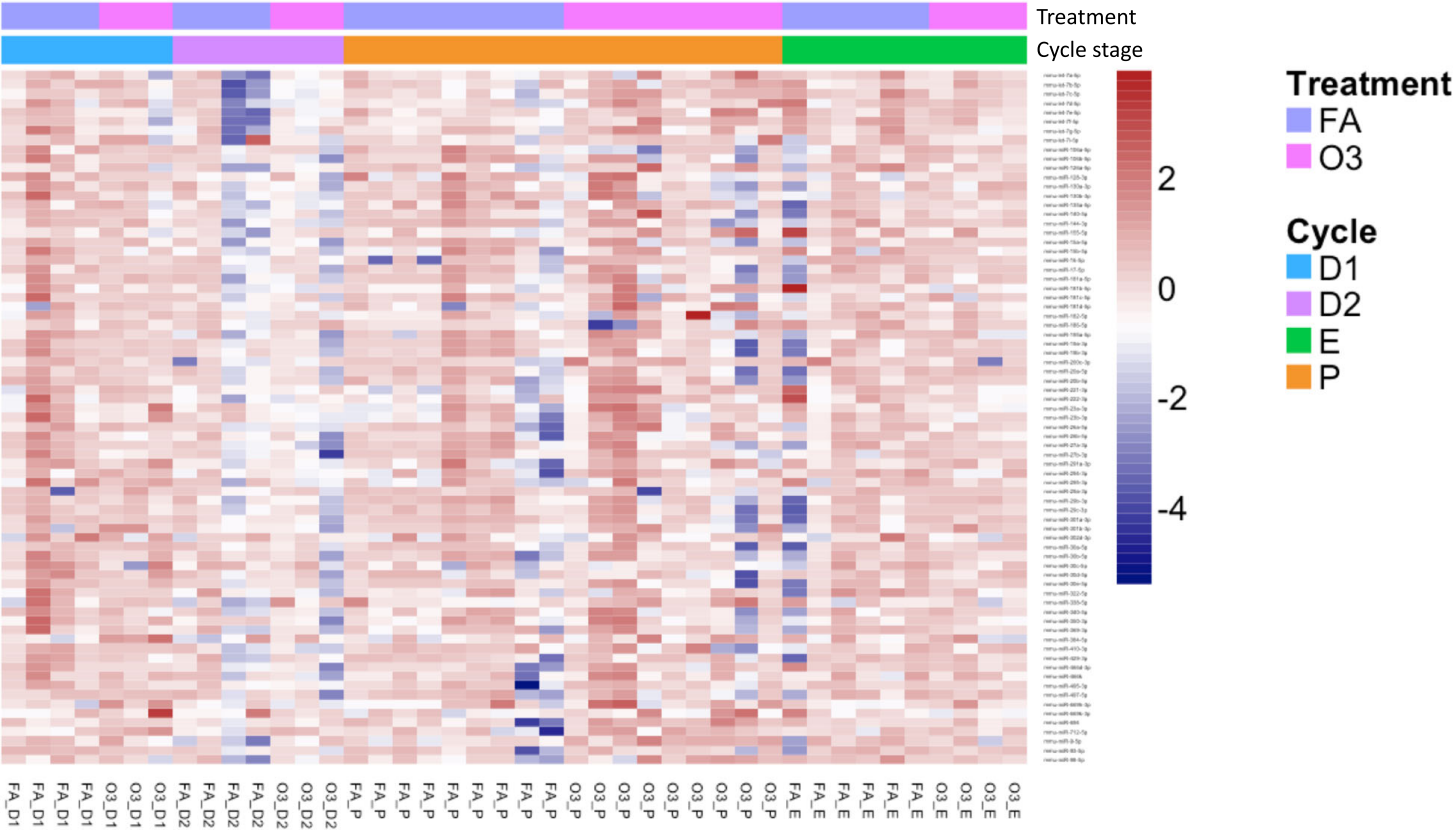
Differential lung miRNA expression in females exposed to O_3_ vs. FA at different stages of the estrous cycle. Cluster analysis of 84 miRNAs measured in lung extracts from female mice exposed to 2 ppm of O_3_ or FA for 3 h. Animals were exposed to O_3_ or FA at different stages of the estrous cycle (D1 = diestrus 1/metestrus, D2 = diestrus 2, P = proestrus, E = estrus). Estrous cycle stage was confirmed by vaginal smear and circulating sex hormone levels. FA filtered air, O3 ozone. Number of mice per group FA_D1 (*n* = 4), FA_D2 (*n* = 4), FA_P (*n* = 9), FA_E (*n* = 6), O3_D1 (*n* = 3), O3_D2 (*n* = 3), O3_P (*n* = 9), O3_E (*n* = 4)

**Fig. 7:**
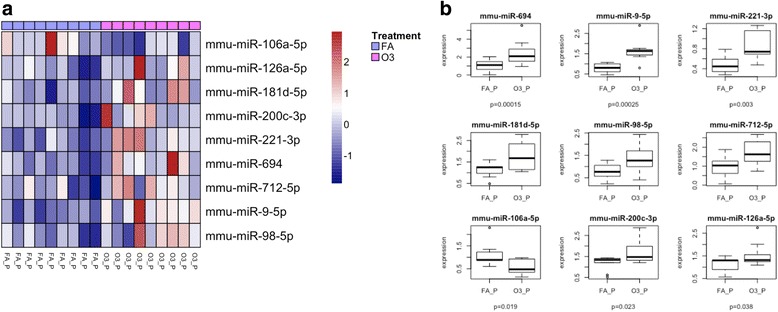
Lung miRNA expression in females exposed to O_3_ vs. FA in proestrus. **a** Cluster analysis of nine miRNAs in lung extracts from female mice exposed to O_3_ or FA in the proestrus stage. **b** Differentially expressed miRNAs in proestrus females exposed to O_3_ vs. FA. FA_P females exposed to filtered air in proestrus (*n* = 9), O3_P females exposed to ozone in proestrus (*n* = 9)

**Fig. 8:**
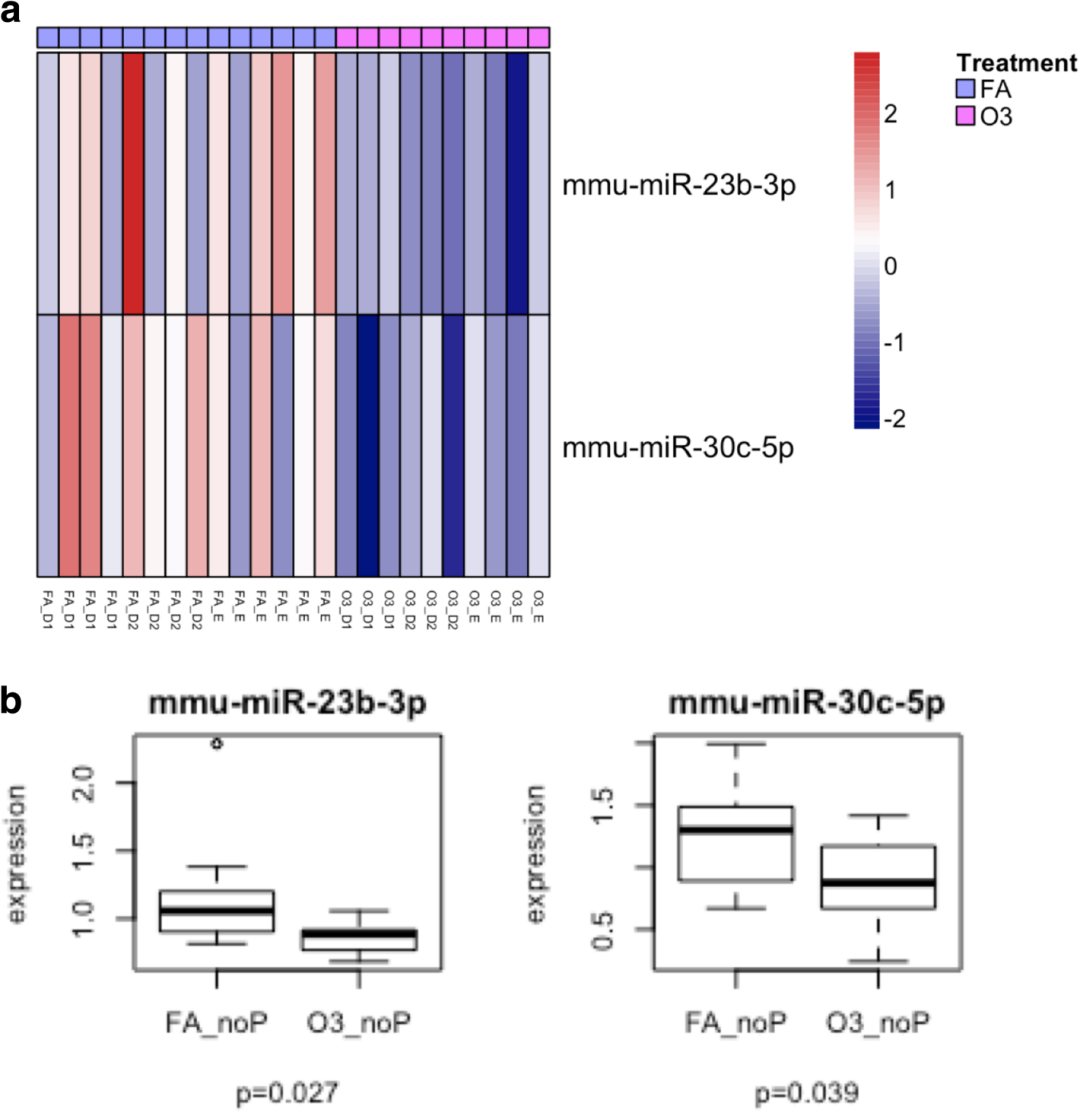
Lung miRNA expression in females exposed to O_3_ vs. FA in non-proestrus cycle stages. **a** Cluster analysis of two inflammatory miRNAs in lung extracts from female mice exposed to O_3_ or FA in metestrus (diestrus 1), diestrus (diestrus 2), or estrus (grouped as “non-proestrus” stages). **b** Differentially expressed miRNAs in non-proestrus females exposed to O_3_ vs. FA. FA_noP females exposed to filtered air in non-proestrus stages (*n* = 14), O3_noP females exposed to ozone in non-proestrus stages (*n* = 10)

**Fig. 9:**
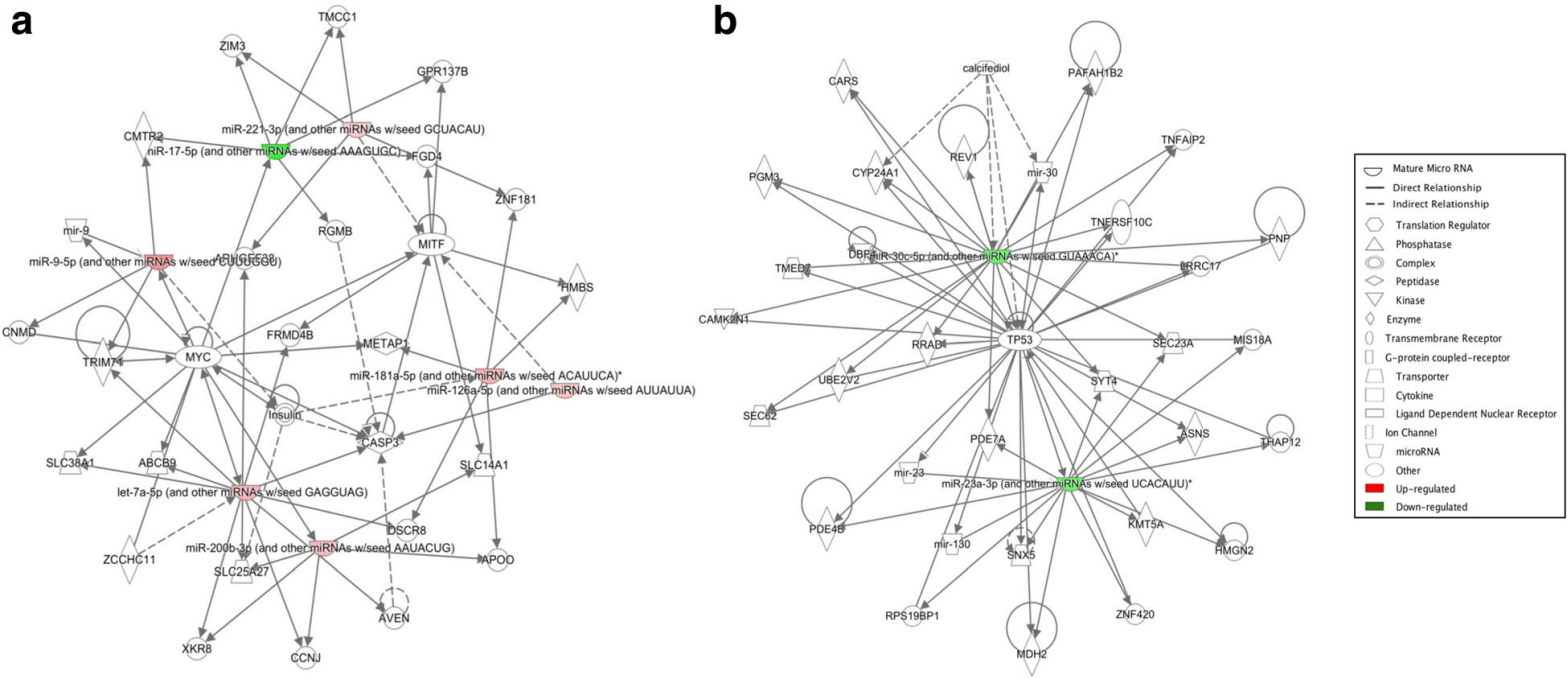
Comparison of networks affected by FA or O_3_ exposure in females at different stages of the estrous cycle. Diagram of biological networks associated with miRNAs whose expression was up- or downregulated in the lungs of animals exposed to FA vs. O_3_ in proestrus (**a**) or non-proestrus stages (**b**). Both diagrams show reported direct (solid lines) and indirect (dashed lines) interactions. Molecules that are downregulated or upregulated are represented as a node in green or red, respectively. Network analysis was performed with Ingenuity Pathway Analysis

Previous observations suggested that the negative effects of air pollution in women’s lung health may be affected by sex hormones. We sought to evaluate whether fluctuations of circulating hormone levels could influence variations in the miRNA response, by exposing female mice to O_3_ or FA at different stages of the estrous cycle. For consistency, experiments were conducted at the same time of the day and harvest of samples was performed at 6:00 pm, to allow for the detection of preovulatory luteinizing hormone and estradiol surges in the evening of proestrus (Table 3) [49]. Our data revealed that there is an influence of the estrous cycle in the miRNA response (Fig. 6). Furthermore, comparison of miRNA expression in females exposed to O_3_ at the proestrus stage vs. all other stages (metestrus, diestrus, or estrus) also revealed differential signatures. Specifically, we found nine differentially expressed miRNAs in females exposed to O_3_ in proestrus: miR-694 (log fold change = 1.492), miR-9-5p (log fold change = 0.836), miR-712-5p (log fold change = 0.667), miR-181d-5p (log fold change = 0.597), miR-98-5p (log fold change = 0.558), miR-200c-3p (log fold change = 0.525), miR-221-3p (log fold change = 0.385), miR-126a-5p (log fold change = 0.421), and miR-106a-5p (log fold change = − 0.527) (Fig. 7). Two out of the eight upregulated miRNAs (miR-712-5p and miR-694) were not associated with any known pathways by IPA. However, according to the literature, miR-712 and miR-694 are molecules associated with key players in lung inflammation such as CCL8, IL-1RAP, IL-7, STAT5a, VEGFA, and BCL6 (Table 4). Comparison of the biological networks affected by O_3_ in the proestrus stage by IPA confirmed differentially affected molecules when compared to the other stages of the estrous cycle. Intriguingly, key players in apoptosis (c-Myc, CASP3) and immune regulators (MITF) were present in the network (Fig. 9). The activation of c-Myc and estrogen have been found to lead into the processing/activation of CASP3, which is highly expressed in the airways when severe lung inflammation occurs [50–52]. In contrast, in females exposed to O_3_ in the metestrus (diestrus 1), estrus, and diestrus 2 stages combined, only two miRNAs were found affected (downregulated): miR-23b-3p (log fold change = − 0.330), and miR-30c-5p (log fold change = − 0.328) (Fig. 8). The main molecular functions associated with these were cellular development, cellular compromise, and cell cycle, but also inflammatory disease (Table 4). Curiously, the tumor suppressor TP53 and TMED7, a protein involved in TLR mediated responses, were present in the molecular analysis, suggesting a correlation with the regulation of miR-23b-3p and miR-30c-5p as well as important mediators of lung immunity such as members of the TNF family (Fig. 9).

## Discussion

Clinical studies have reported differential outcomes for lung disease in men vs. women, as well as an increased susceptibility for women to the damaging effects of air pollution. Despite this evidence, the associated mechanisms of the pollution-induced inflammatory response in the male and female lung remain unknown. In our previous work, we reported sex-specific expression of inflammatory mediators in response to O_3_ exposure and a potential role of circulating hormone levels in the control of cytokine expression and associated intracellular pathways [35, 36]. In the current study, we have further characterized sex-specific miRNA signatures in the lungs of male and female mice activated in response to O_3_ exposure and contributions of the estrous cycle to this regulation, since miRNAs have been previously reported as key regulators of oxidative stress responses in various tissues and diseases [53]. Our analysis using PCR arrays revealed multiple trends towards differences in miRNA expression that, although not strictly statistically significant due to lack of power, provide useful information on biological pathways affected in the male and female lung. Importantly, our results show differences in miRNA expression and differential activation of regulatory pathways in the lungs of male and female mice exposed to both FA and O_3_. Moreover, we found differences in the lung miRNA profiles of female mice exposed to O_3_ at different stages of the estrous cycle. Together, our data indicate that both sex and hormonal status can influence lung miRNA expression and, therefore, regulation of inflammatory genes, in response to O_3_ exposure.

The lung expresses both estrogen and progesterone receptors, and these control multiple functions of the organ [54, 55]. Several studies, including ours, have hypothesized that female sex hormones can act as physiological modulators of lung function and immunity, via inflammatory gene expression regulation [36, 56, 57]. Evidence from clinical studies reporting menstrual cycle-dependent asthma exacerbations in women and variations in respiratory disease clinical outcomes with pregnancy and oral contraceptive use are in agreement with this hypothesis [58, 59]. A potential mechanism by which sex hormones can effectively affect gene expression is through modulation of inflammatory gene expression by miRNAs. While other studies have explored the contributions of sex hormones to the miRNome [60], to our knowledge, this is the first study reporting sex-specific and estrous cycle day-specific miRNA profiles in response to O_3_ exposure. Our results also showed that the lungs of male and female mice express different miRNA profiles under basal conditions, suggesting specific roles for these miRNAs in the male and female lung.

Comparison of basal miRNA expression in males vs. females revealed two miRNAs that were differentially expressed, miR-222-3p and miR-466k. Most of the predicted gene networks affected by these miRNAs were associated with cellular growth, proliferation, and cancer. However, miR-466k is downregulated in females exposed to O_3_. The miR-466 family affects apoptosis regulation in mammalian cells and is a master regulator of several pathways associated with regulatory T cell development and function [61, 62]. In response to O_3_, both groups had a total of eight miRNAs that were differentially expressed. There were several similarities and differences between the groups in terms of gene networks affected. The group of miRNAs upregulated in the lungs of female mice exposed to O_3_ was associated with important inflammatory pathways such as the IL-10 and SOCS families. On the other hand, most miRNAs differentially expressed in male mice exposed to O_3_ were linked to the IL-6 family. Together, these results suggest that O_3_ induces unique molecular signatures and miRNA expression profiles in the male and female lung, contributing to the previously reported sex differences in inflammatory gene expression and lung immune function.

Of particular interest is the involvement of miR-712 in the regulation of the immune response and O_3_-induced lung inflammation. While IPA did not associate any regulatory pathways to this miRNA, there is an apparent increase in the expression of this miRNA in females when compared to males exposed to O_3_. In addition, previous studies have shown that miR-712 downregulates a tissue inhibitor of metalloproteinase 3 (TIMP3), which in turn activates matrix metalloproteinases 2 and 9 (MMP2, MMP9), as well as a disintegrin and metalloproteases 10 and 17 (ADAM10, ADAM17) [40]. These metalloproteinases stimulate inflammation and, therefore, the expression of cytokines such as IL-6 and its receptor (IL6R), which according to our previous studies are highly expressed in the lungs of female mice exposed to O_3_ [35]. In addition, our data showed high expression of let-7i-5p in males, but not females, exposed to O_3_. Interestingly, this miRNA is known for inhibiting IL-6 expression, which levels are significantly higher in lung tissue from females vs. males exposed to O_3_ [36].

The comparison of miRNA expression in female mice exposed to FA vs. O_3_ revealed upregulation of both miR-9-5p and miR-130a-3p, which are known for targeting SOCS5 and altering macrophage polarization, respectively [63, 64]. The significance of these findings relies on the fact that these targeted molecules are involved in T cell differentiation and that it has been suggested that the susceptibility of female mice to O_3_ may be due to a Th1/Th2 imbalance [65]. Our results also showed that miR-106a-5p was upregulated in males exposed to O_3_ but downregulated in females exposed to O_3_ in the proestrus stage. It has been shown that miR-106a-5p targets interleukin-10 (IL-10), an anti-inflammatory cytokine that is defective in many inflammatory diseases including asthma and allergic lung inflammation [66]. Intriguingly, knockdown of this miRNA in an established allergic airway inflammation significantly alleviated most of the features of asthma such as airway hyperresponsiveness, increased Th2 response, and sub-epithelial fibrosis, along with increased IL-10 levels in the lungs of male mice [67]. However, the role of miR-106a-5p and its relationship with sex hormones and environmental pollutants remains unexplored in the female lung.

More recently, environmental factors such as O_3_ and airborne particulate matter have been linked to altered miRNA expression, suggesting that miRNAs may be involved in the adverse health effects of air pollution exposure [68]. Our results suggest a link between miRNAs and top diseases such as cancer and endocrine disorders. In the particular case of lung cancer, several studies have showed that certain miRNA profiles classified lung cancer subtypes and that specific miRNA expression signatures associated with lung cancer prognosis [69]. Both miR-221 (highly expressed in the proestrus stage of females exposed to O_3_) and miR-222 (upregulated in males exposed to O_3_) are involved in the development and progression of lung cancer by targeting the tumor suppressor genes PTEN and TIMP3 [70]. Moreover, overexpression of miR-221/222 is known to inhibit apoptosis and promote cell migration by downregulating PTEN and TIMP3 [71]. More importantly, miR-221/222 has been reported to target estrogen receptor alpha (ESR1), and miR-221-3p has been shown to regulate IL-6 release from abnormal airway smooth muscle in patients with severe asthma, especially women [72, 73]. Finally, we found that miR-23b-3p was downregulated in females exposed to O_3_ in non-proestrus stages (i.e., when estrogen levels are low) but not in females exposed in proestrus. To this end, studies have shown that miR-23b inhibits TGF-β1-induced airway smooth muscle proliferation and promotes apoptosis, indicating a potential role of this miRNA in lung functions and diseases that are affected by the menstrual cycle [74–77].

In summary, our studies presented here revealed sex-specific miRNA expression networks in the lungs of mice exposed to O_3_ or FA. Major differences involved pathways linked to the inflammatory response, endocrine diseases, respiratory function, and cancer. In addition, we identified an estrous cycle-dependent miRNA signature in females exposed to O_3_. Interestingly, more miRNAs were affected in females exposed to the air pollutant in the proestrus stage of the cycle (i.e., when circulating hormone levels are high) vs. the rest of the stages, indicating that sex hormones could potentially contribute to the immune response to air pollution via regulation of miRNAs. Future studies using ovariectomy and hormone replacement prior to O_3_ exposure could help elucidate the mechanisms behind this differential expression.

## Conclusion

Using a mouse model, we found differential activation of miRNA regulatory networks in males vs. females in response to O_3_ exposure. Our data revealed that both sex and hormonal status can influence the lung miRNA response to O_3_. We also found altered expression of miRNAs that have been previously associated with IL-6 regulation in response to O_3_, in females and males, as well as sex differences in their expression levels. Together, these results indicate that sex-specific miRNA regulation of inflammatory gene expression could mediate differential health outcomes in men and women exposed to air pollution. This information can have significant implications for environmental health and help in the development of novel sex/gender-specific therapeutics to treat and prevent lung disease.

## Additional files

Additional file 1: **Figure S1.** Sex differences in inflammatory miRNA expression. A. Cluster analysis of 84 inflammatory miRNAs in lung extracts from male and female mice. B. Individual expression of miRNAs differentially expressed in lung tissue from males vs. females. M, males (*n* = 6); F females (*n* = 23). FA_M males exposed to filtered air, FA_D1 females exposed to filtered air in diestrus 1, FA_D2 females exposed to filtered air in diestrus 2, FA_P females exposed to filtered air in proestrus, FA_E females exposed to filtered air in estrus. (PDF 320 kb)

Additional file 2: **Table S1.** Target genes and associated pathways for differentially expressed miRNAs in lung tissue of unexposed male and female mice. (DOCX 23 kb)

Additional file 3: **Figure S2.** Sex differences in networks affected by differentially expressed miRNAs. Diagram of biological networks affected by differentially expressed miRNAs in the lungs of male and female animals exposed to FA (A) of O_3_ (B). Both diagrams show reported direct (solid lines) and indirect (dashed lines) interactions. Molecules that are downregulated or upregulated are represented as a node in green or red, respectively. Network analysis was performed with Ingenuity Pathway Analysis. (PDF 374 kb)

## Abbreviations

ADAM10: A disintegrin and metalloproteases 10
ADAM17: A disintegrin and metalloproteases 17
COP: Chronic obstructive pulmonary disease
D1: Diestrus 1/metestrus
D2: Diestrus
E: Estrus
FA: Filtered air
FA_D1: Females exposed to filtered air in diestrus 1
FA_D2: Females exposed to filtered air in diestrus 2
FA_E: Females exposed to filtered air in estrus
FA_M: Males exposed to filtered air
FA_noP: Females exposed to filtered air in non-proestrus stages
FA_P: Females exposed to filtered air in proestrus
FDR: Benjamini-Hochberg false discovery rate
IACUC: Institutional Animal Care and Use Committee
IL-10: Interleukin-10
IL-6: Interleukin-6
miRNAs: MicroRNAs
MMP2: Matrix metalloproteinases 2
MMP9: Matrix metalloproteinases 9
O_3_: Ozone
O3_D1: Females exposed to ozone in diestrus 1
O3_D2: Females exposed to ozone in diestrus 2
O3_E: Females exposed to ozone in estrus
O3_M: Males exposed to ozone
O3_noP: Females exposed to ozone in non-proestrus stages
O3_P: Females exposed to ozone in proestrus
P: Proestrus
ROS: Reactive oxygen species
TIMP3: Tissue inhibitor of metalloproteinase 3
