# Supplementary material for: Sex-specific microRNA expression networks in an acute mouse model of ozone-induced lung inflammation
Source: Biol Sex Differ. 2018 May 8;9:18. doi: 10.1186/s13293-018-0177-7 (PMC5941588; doi:10.1186/s13293-018-0177-7)
Supplement: Supplementary file 1 — Figure S1. Sex differences in inflammatory miRNA expression. A. Cluster analysis of 84 inflammatory miRNAs in lung extracts from male and female mice. B. Individual expression of miRNAs differentially expressed in lung tissue from males vs. females. M, males (n = 6); F females (n = 23). FA_M males exposed to filtered air, FA_D1 females exposed to filtered air in diestrus 1, FA_D2 females exposed to filtered air in diestrus 2, FA_P females exposed to filtered air in proestrus, FA_E females exposed to filtered air in estrus. (PDF 320 kb) [file 13293_2018_177_MOESM1_ESM.pdf]

A.

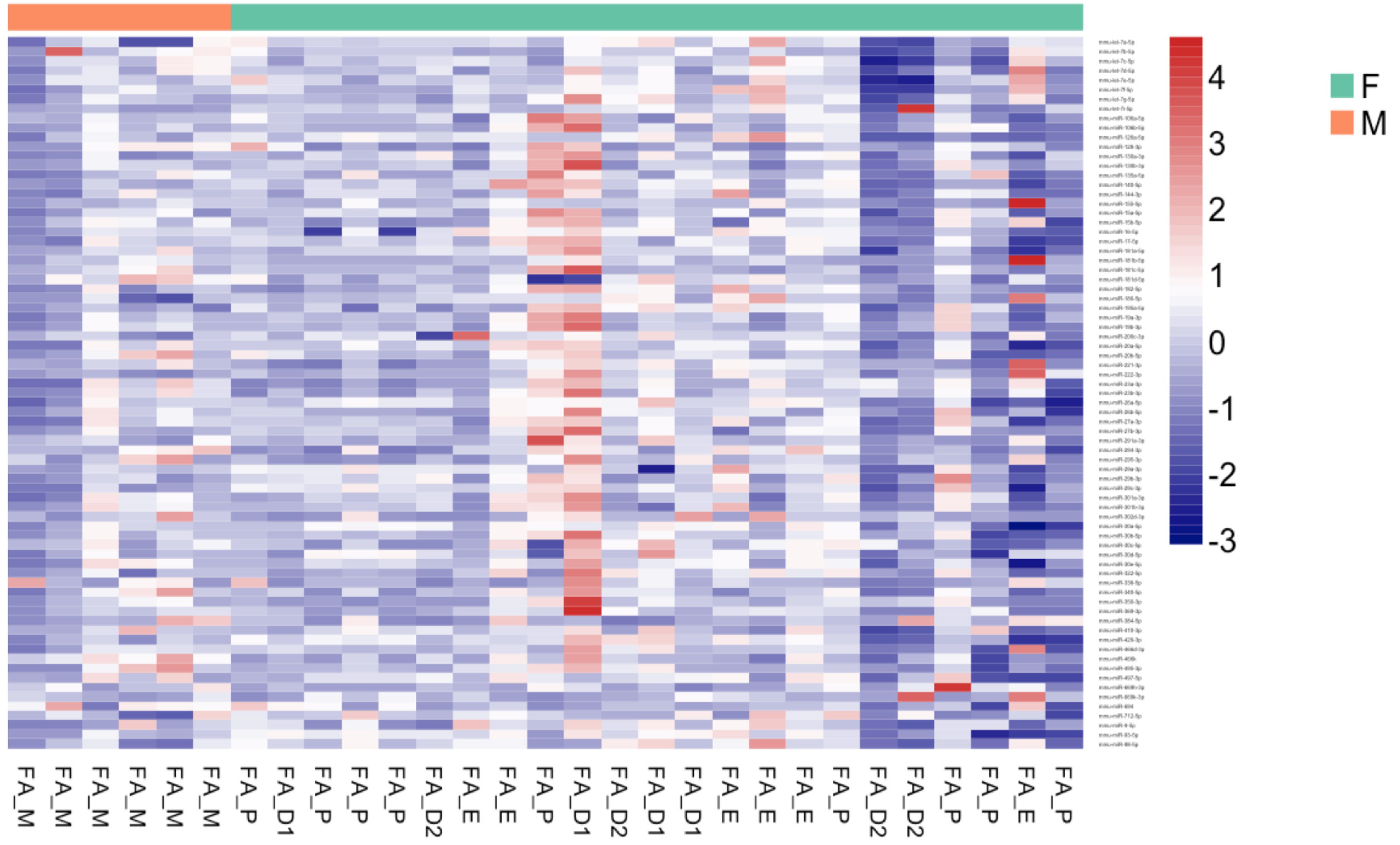
F
M
4
3
2
1
0
-1
-2
-3
FA_M
FA_M
FA_M
FA_M
FA_M
FA_M
FA_P
FA_D1
FA_P
FA_P
FA_P
FA_D2
FA_E
FA_E
FA_P
FA_D1
FA_D2
FA_D1
FA_D1
FA_E
FA_E
FA_E
FA_P
FA_D2
FA_D2
FA_P
FA_P
FA_E
FA_P

B.

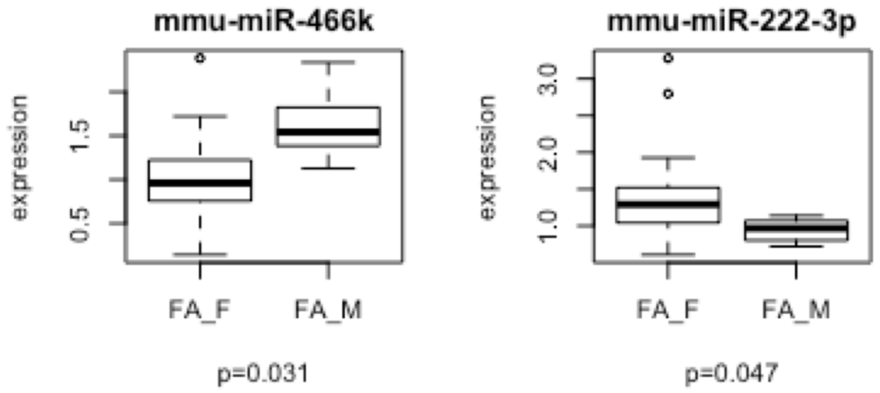
mmu-miR-466k
expression
0.5
1.5
FA_F
FA_M
p=0.031
mmu-miR-222-3p
expression
1.0
2.0
3.0
FA_F
FA_M
p=0.047
