# Supplementary material for: Sex-specific microRNA expression networks in an acute mouse model of ozone-induced lung inflammation
Source: Biol Sex Differ. 2018 May 8;9:18. doi: 10.1186/s13293-018-0177-7 (PMC5941588; doi:10.1186/s13293-018-0177-7)
Supplement: Supplementary file 2 — Table S1. Target genes and associated pathways for differentially expressed miRNAs in lung tissue of unexposed male and female mice. (DOCX 23 kb) [file 13293_2018_177_MOESM2_ESM.docx]

**Table S1. Target genes and associated pathways for differentially expressed miRNAs in lung tissue of unexposed male and female mice**

| 1. *Genes targeted by differentially expressed miRNAs* | | | | | | | | |
| --- | --- | --- | --- | --- | --- | --- | --- | --- |
| AGO2  ARID3B  BBC3  BCL2  BCL2L11 | BDNF  BEAN1  BRAF, CCND1  CDKN2B | CLVS2  E2F1  ESR1  FBXO47, FHL2 | | FOS  FOXO3  FOXP3  JUN  KIT | | PTEN  RORB  SBK1  TIMP3  TP53 | | TSC22D3  UBQLN2  VAPB |
| 1. *Differences in top diseases and bio-functions* | | | | | | | | |
| Diseases and Disorders | | | | | *P* Value | | | |
| Cancer | | |  | |  | | 5.00E-02 - 9.36E-05 | |
| Connective tissue disorders | | |  | |  | | 1.87E-04 - 9.36E-05 | |
| Organismal injury and abnormalities | | |  | |  | | 5.00E-02 - 9.36E-05 | |
| 1. *Top molecular and cellular functions* | | | | | | | | |
| Molecular and Cellular Functions  *P* Value | | | | | | | | |
| Cell-to-cell signaling and interaction 2.48E-03 - 1.40E-04 | | | | | | | | |
| Cellular growth and proliferation 1.20E-02 - 2.34E-04 | | | | | | | | |
| Cell death and survival 1.68E-02 - 3.74E-04 | | | | | | | | |
| 1. *Top physiological system development and function* | | | | | | | | |
| Development and Function *P* Value | | | | | | | | |
| Hematological system development and function 6.55E-04 - 6.55E-04 | | | | | | | | |
| Tissue morphology 1.92E-03 - 1.92E-03 | | | | | | | | |
| Connective tissue development and function 2.53E-03 - 2.48E-03 | | | | | | | | |
| *E. Top associated network functions* | | | | | | | | |
| Associated Network Functions | | |  | | | | Score | |
| Cellular growth and proliferation, gene expression, cancer | | | | | | | 6 | |
