# Supplementary figures and images for: Sex-specific microRNA expression networks in an acute mouse model of ozone-induced lung inflammation
Source: Biol Sex Differ. 2018 May 8;9:18. doi: 10.1186/s13293-018-0177-7 (PMC5941588; doi:10.1186/s13293-018-0177-7)

A.

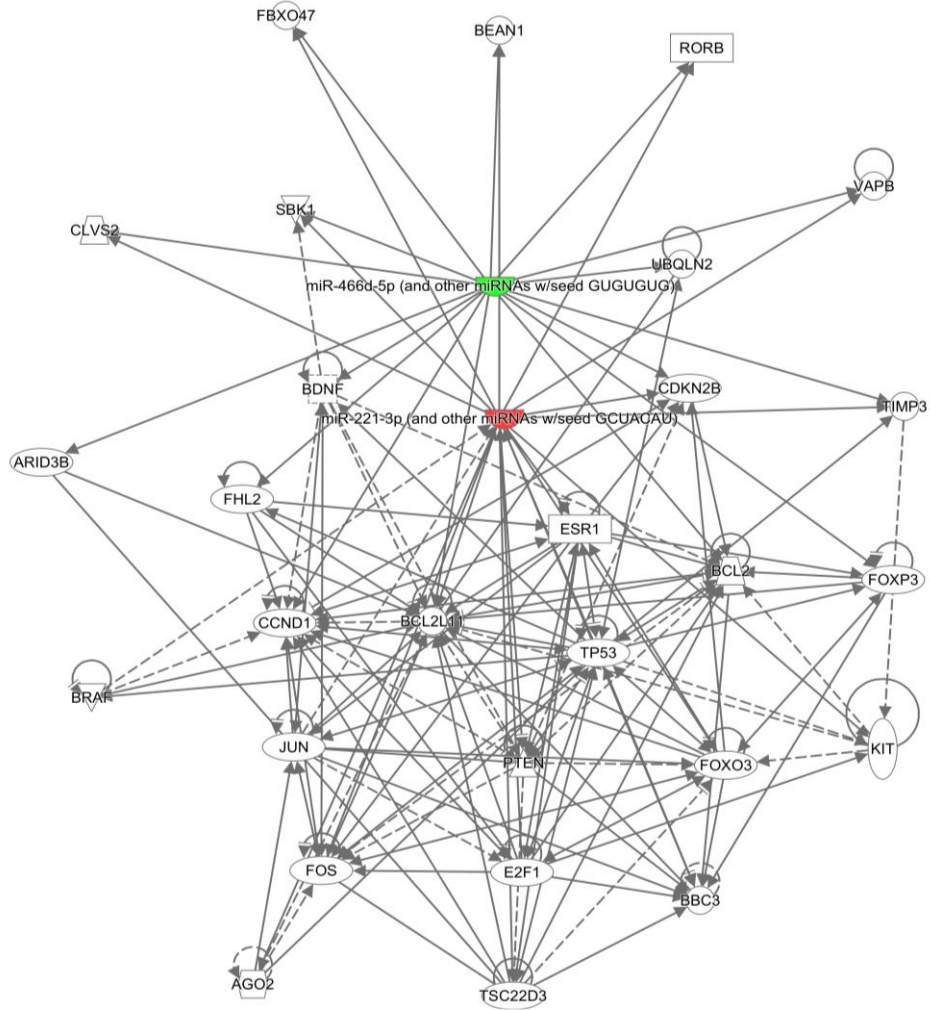

B.

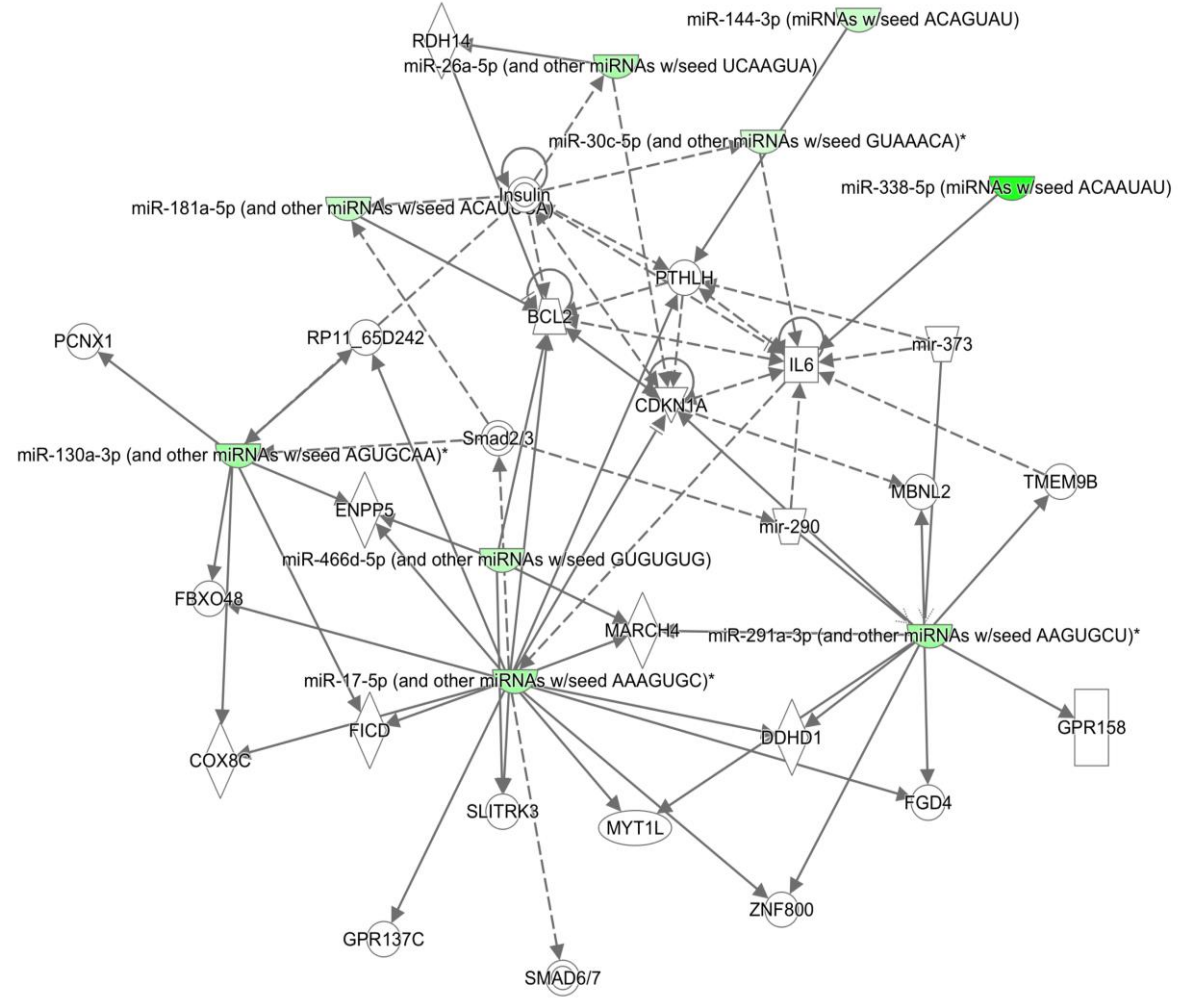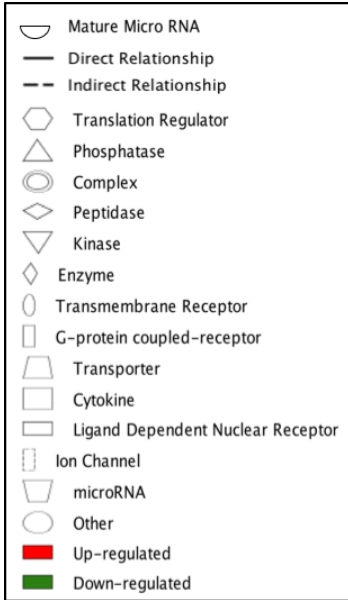

Supplement: Supplementary file 3 — Figure S2. Sex differences in networks affected by differentially expressed miRNAs. Diagram of biological networks affected by differentially expressed miRNAs in the lungs of male and female animals exposed to FA (A) of O3 (B). Both diagrams show reported direct (solid lines) and indirect (dashed lines) interactions. Molecules that are downregulated or upregulated are represented as a node in green or red, respectively. Network analysis was performed with Ingenuity Pathway Analysis. (PDF 374 kb) [file 13293_2018_177_MOESM3_ESM.pdf]
